# The contribution of periaqueductal gray in the regulation of physiological and pathological behaviors

**DOI:** 10.3389/fnins.2024.1380171

**Published:** 2024-04-08

**Authors:** Hui Zhang, Zhe Zhu, Wei-Xiang Ma, Ling-Xi Kong, Ping-Chuan Yuan, Li-Fang Bu, Jun Han, Zhi-Li Huang, Yi-Qun Wang

**Affiliations:** ^1^Department of Pharmacology, School of Basic Medical Sciences, State Key Laboratory of Medical Neurobiology, Institutes of Brain Science and Collaborative Innovation Center for Brain Science, Joint International Research Laboratory of Sleep, Fudan University, Shanghai, China; ^2^Anhui Provincial Engineering Laboratory for Screening and Re-evaluation of Active Compounds of Herbal Medicines in Southern Anhui, Anhui Provincial Engineering Research Center for Polysaccharide Drugs, Wannan Medical College, Wuhu, China; ^3^Department of Anesthesiology, Zhongshan Hospital, Fudan University, Shanghai, China

**Keywords:** periaqueductal gray, pain modulation, defensive behavior, predatory hunting, sleep-wake states

## Abstract

Periaqueductal gray (PAG), an integration center for neuronal signals, is located in the midbrain and regulates multiple physiological and pathological behaviors, including pain, defensive and aggressive behaviors, anxiety and depression, cardiovascular response, respiration, and sleep-wake behaviors. Due to the different neuroanatomical connections and functional characteristics of the four functional columns of PAG, different subregions of PAG synergistically regulate various instinctual behaviors. In the current review, we summarized the role and possible neurobiological mechanism of different subregions of PAG in the regulation of pain, defensive and aggressive behaviors, anxiety, and depression from the perspective of the up-down neuronal circuits of PAG. Furthermore, we proposed the potential clinical applications of PAG. Knowledge of these aspects will give us a better understanding of the key role of PAG in physiological and pathological behaviors and provide directions for future clinical treatments.

## 1 Introduction

Periaqueductal gray (PAG) is located in the midbrain and is the main structure involved in integrating aversion information and reaction output in defensive and emotional behaviors (Vázquez-León et al., 2023). This region has rich and diverse functional characteristics, which are essential for the survival and reproduction of mammals. Some of the main functions of this region include defense and aggressive behavior, fear, anxiety, pain, and dyspnea, as well as the regulation of the corresponding cardiovascular changes (George et al., 2019). According to the functional characteristics and anatomical location, PAG can be divided into four longitudinal columns: dorsomedial PAG (DMPAG), dorsolateral PAG (DLPAG), lateral PAG (LPAG), and ventrolateral PAG (VLPAG). Dorsal PAG (DPAG, which includes DMPAG and DLPAG) is mainly involved in active defense, aggressive behavior, tachycardia, elevated blood pressure (BP), and other related reactions. VLPAG is mainly involved in passive defense behaviors, opioid-mediated deep analgesia and sleep regulation. For example, L/DLPAG acts on avoidable stressors, showing evasive threat responses, such as “flight” and “fight,” enhancing connections with processing emotional regions and actively responding to stressors. As a result, humans respond quickly to acute injurious stimuli and avoid danger. VLPAG plays a role in behavioral responses to unavoidable stressors, exhibiting “freezing”-related behaviors. During certain perceived threats, including the perception of greater dyspnea, VLPAG is less connected to sensorimotor structures, which is manifested as reduced activity in patients (Benarroch, 2012; Faull and Pattinson, 2017). PAG receives projections from multiple regions of the forebrain and integrates the information to specific regions, such as pons-medulla, to mediate active and passive responses. In recent years, detailed studies of various subregions of PAG in human beings have improved our understanding of the functional characteristics, neural loop, and molecular mechanism of PAG. Next, we will summarize the functional characteristics of the complex neural network of PAG and the related neurotransmitters.

## 2 Anatomical structure and neural connections of PAG

PAG is a longitudinally organized structure surrounding the mesencephalic aqueduct in the midbrain. It stretches along the caudal-rostral axis from the posterior commissure to the locus coeruleus (LC) (Mokhtar and Singh, 2023). The lateral boundaries of PAG are defined by fibers from the mesencephalic trigeminal tract and tectospinal tract, which originate from the deep layers of the superior colliculus (Faull et al., 2019). A recent study utilizing single-nucleus RNA sequencing and multiplexed error-robust fluorescence *in situ* hybridization has provided novel molecular and functional insights into the PAG and surrounding regions (Vaughn et al., 2022). Researchers have identified 144 neuron subpopulations and classified them into 19 metaclusters based on their spatial motifs, refining the previous coarse anatomical subdivisions of PAG across its column and opening new avenues for a mechanistic understanding of the PAG function (Vaughn et al., 2022).

**FIGURE 1:**
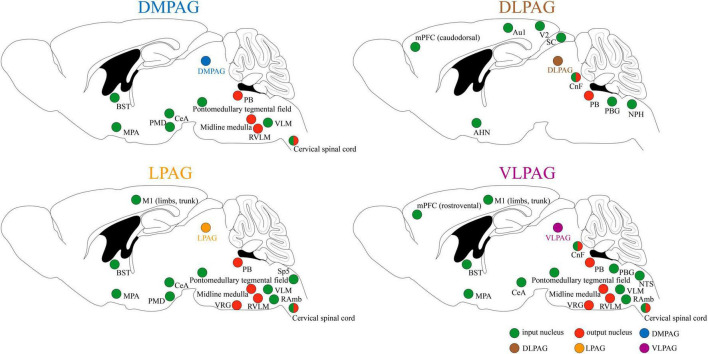
Input and output of neuronal circuits in different subregions of PAG. Au1, primary auditory cortex; AHN, anterior hypothalamic area; BST, bed nucleus of the stria terminalis; CeA, central amygdala; CnF, cuneiform nucleus; DMPAG, dorsomedial periaqueductal gray; DLPAG, dorsolateral periaqueductal gray; LPAG, lateral periaqueductal gray; MPA, medial preoptic area; mPFC, medial prefrontal cortex; M1, primary motor cortex; NPH, nucleus praepositus hypoglossi; NTS, nucleus tractus solitarius; PMD, dorsal premammillary nucleus; PB, parabrachial complex; PBG, periparabigeminal nucleus; RVLM, rostral ventrolateral medulla; RAmb, retroambiguus nucleus; SC, superior colliculus; Sp5, spinal trigeminal nucleus; VLPAG, ventrolateral periaqueductal gray; VLM, ventrolateral medulla; V2, secondary visual cortex; VRG, ventral respiratory group.

Because of its extensive connections with the cortex and various brainstem nuclei, PAG serves as a critical midbrain hub for sensory-motor integration (see Figure 1 for details; Zare et al., 2019; Schottelkotte and Crone, 2022; Ma et al., 2023). It primarily receives input from regions such as the medial prefrontal cortex (mPFC), central amygdala (CeA), anterior cingulate cortices (ACC), bed nucleus of the stria terminalis (BST), hypothalamus, and dorsal premammillary nucleus (PMD), allowing for the relay and integration of sensory information from diverse modalities (Benarroch, 2012; Faull et al., 2019; Zare et al., 2019; Schottelkotte and Crone, 2022). In turn, the PAG conveys these integrated signals by projecting efferent connections to brainstem regions, including the parabrachial complex (PB), midline medulla, rostral ventrolateral medulla (RVLM), and cervical spinal cord (SC) (Benarroch, 2012; Faull et al., 2019; Zare et al., 2019). This enables the PAG to orchestrate and modulate instinctive behaviors, including pain modulation, respiration, cardiovascular responses, vocalization, crying, coughing, micturition, and motor actions, through downstream pathways (Benarroch, 2012; Schottelkotte and Crone, 2022).

## 3 Main functional characteristics and mechanisms of PAG

### 3.1 Double regulation of pain by the PAG and its mechanism

Functioning as a central hub for processing ascending and descending pain signals, the PAG primarily interacts with the rostral ventromedial medulla (RVM) to control pain signals at SC level (Kim et al., 2018; Chung et al., 2020). The PAG-RVM pathways project to pain-transmitting neurons in the dorsal horn of the SC and the trigeminal nucleus caudalis, enabling bidirectional nociception control (Kim et al., 2018). This allows the PAG to both facilitate and suppress pain processing, which is influenced by various behavioral, emotional, and pathological factors (Benarroch, 2012). Early research primarily focused on the involvement of PAG in the ascending pain pathway, with studies dating back to Magoun et al. (1937) revealing its role in pain and vocalization. Further investigations, such as those conducted by Melzack et al. (1958) on cats, confirmed that lesions in the VLPAG significantly reduce pain perception. Human studies have also shown that stimulating the PAG region can evoke sensations of pain, vibration, eye movement, and fear (Nashold et al., 1969).

Since the 1960s, the involvement of PAG in analgesia has been acknowledged, as electrical stimulation of PAG has been found to produce profound analgesia in male rats (Reynolds, 1969). Notably, short-term noxious skin stimuli activate the LPAG and DPAG to trigger not only sympathetic excitations but also transient, non-opioid-mediated analgesia (Keay and Bandler, 2001; Lumb, 2004). Conversely, deep somatic pain, visceral pain, or repeated superficial pain activates the VLPAG, leading to long-lasting opioid-dependent analgesia associated with vascular inhibition and immobility behaviors (Bandler et al., 2000). PAG and its descending circuit exhibit sexual dimorphism, leading to different responses to pain and analgesia between males and females. For example, activation of dopamine (DA) neurons in the VLPAG/dorsal raphe (DR) and BST promotes antinociception in male mice but induces locomotion in female mice (Yu W. et al., 2021). In female rats, despite having greater PAG connections to RVM, morphine and pain induce less activation of PAG-RVM projecting neurons compared with male rats, resulting in reduced pain reduction upon microinjection of morphine into the PAG (Loyd et al., 2007; Linnman et al., 2012). Moreover, persistent inflammatory pain upregulates cannabinoid receptor 1 expression in the PAG, contributing to sex-specific differences in pain modulation, with males exhibiting a greater enhancement in pain-induced G-protein activation than females (Wilson-Poe et al., 2021). These findings reflect the intricate nature of pain modulation mediated by the PAG across genders.

Various types of neurotransmitters in the PAG, including glutamate, γ-aminobutyric acid (GABA), opioid, cannabinoid, DA, and serotonin (5-HT), participate in fine-tuning the balance between descending facilitation and descending inhibition of nociception. Glutamate or ionotropic glutamate receptor agonists microinjected into the VLPAG elevate sensory thresholds, while glutamatergic antagonists induce hyperalgesia (Samineni et al., 2017; Nguyen et al., 2023). On the contrary, injection of GABA agonists into the VLPAG produces hyperalgesia while injection of its antagonists produces antinociceptive effects (Samineni et al., 2017; Nguyen et al., 2023). Moreover, researchers have identified eight subtypes of glutamate metabotropic receptors (mGluR1–8) in the PAG, of which mGluR1 and mGluR5 cause hyperalgesia and mGluR2–4 and mGluR6–8 induce analgesia (Peng et al., 2023). With regard to opioids, injecting μ-opioid receptor agonists into the VLPAG generates antinociception, with opioids primarily inducing analgesia via presynaptic inhibition of GABAergic and 5-HTergic projections to the RVM (Vázquez-León et al., 2023). Cannabinoid receptor 1, which is broadly expressed in both the DLPAG and VLPAG, activates the descending pain modulatory circuit. Their activation in the VLPAG induces antinociception and anti-hyperalgesia, while their activation in the DLPAG mediates opioid-independent stress-induced analgesia (Bouchet and Ingram, 2020). Additionally, studies have shown that activating 5-HT receptors in the PAG induces analgesia and contributes to fear-induced antinociception (Vázquez-León et al., 2023). In addition to the previously mentioned neurotransmitters, other substances play vital roles in pain modulation within the PAG. For example, prostaglandins within the PAG have pronociceptive actions (Drake et al., 2016), and a subpopulation of parvocellular oxytocin neurons projecting to the VLPAG has been found to mediate analgesia through the PAG-controlled descending pain modulatory system (Iwasaki et al., 2023). Additionally, melatonin acts through melatonin receptor 2 in the VLPAG to induce analgesia (Lopez-Canul et al., 2015). However, the analgesic effects in the PAG can produce certain side effects, with glutamatergic or GABAergic neurons in the VLPAG being involved in the regulation of fear and anxiety responses (Morgan et al., 2008; Tovote et al., 2016). Nevertheless, recent research has found that activation of specific DA neurons in the PAG solely produces analgesia without anxiety-like behaviors (Taylor et al., 2019). These findings highlight the complexity of pain modulation in the PAG and provide potential avenues for understanding and managing pain-related responses and ailments.

### 3.2 PAG-induced defensive responses and the underlying neurochemical mechanisms

Previous studies have elucidated the pivotal role of PAG in generating innate defensive responses. PAG stimulation in rats, cats, and mice using electrical, chemical, and optogenetic methods has been found to elicit defensive reactions (Deng et al., 2016). Notably, different columns within the PAG play distinct roles in modulating defensive behaviors. Extensive stimulation and lesion experiments have revealed that DLPAG is primarily associated with active coping responses, including vocalization and vigorous escape, whereas VLPAG is primarily involved in passive coping responses, such as tonic immobility (Motta et al., 2017; Vieira-Rasteli et al., 2018). Recent research has further shed light on the neuronal coding differences between flight and freezing behaviors within the DPAG, revealing that neural activity is more intense during flight behavior, characterized by higher firing rates compared with freezing behavior (Liu et al., 2022). Additionally, recent studies have highlighted the significance of PAG in mediating conditioned defensive responses. For instance, optical inhibition of VLPAG glutamate neurons reduces freezing in the conditioning context (Tovote et al., 2016), while injections of N-methyl-D-aspartate (NMDA) and corticotropin-releasing factor into the DPAG enhance freezing in response to a fear-conditioned context (Reis et al., 2021). There is evidence indicating that innate-freezing and learned-freezing behaviors may be regulated by the DPAG and VLPAG, respectively (Borelli et al., 2005; Isosaka et al., 2015).

Neurotransmitters such as glutamate, GABA, 5-HT, and endogenous opioids are present within the PAG, where they interact in a complex manner to regulate defensive responses. Manipulation of these neurotransmitter systems within the PAG has been shown to influence defensive behaviors. For instance, injection of semicarbazide, an inhibitor of glutamate dehydrogenase, into the DLPAG induces immobility behavior, while administration of bicuculline, a GABA-A receptor antagonist, in the same area elicits significant escape responses (Borelli et al., 2005). Activation of the 5-HT1A and μ-opioid receptors within the DPAG has been found to increase the threshold electrical current intensity required for evoking escape (Roncon et al., 2017). Moreover, injections of cannabinoid receptor agonists into the DLPAG decrease freezing behavior associated with contextual fear (Resstel et al., 2008). These findings emphasize the intricate interplay between neurotransmitters and their receptors in the PAG, highlighting their significant contributions toward modulating defensive behaviors.

PAG receives afferents from various structures known to be activated by threats, including amygdala, hypothalamus, and premammillary nucleus (Hadjipavlou et al., 2006; Pernía-Andrade et al., 2021; Wang et al., 2021a,b). These inputs play critical roles in modulating defensive behaviors. Recent studies have revealed that the cholecystokinin-expressing hypothalamic PMD cells projecting to the DLPAG control escape behavior induced by a range of innate threats (Wang et al., 2021a). Additionally, the fastigial nucleus-VLPAG pathway has been found to regulate learned defensive responses by modulating the association between conditioned and unconditioned stimuli, thus influencing memory formation (da Silva et al., 2023).

### 3.3 PAG plays a vital role in the initiation, execution, and coordination of predatory hunting

Predatory hunting is an innate and conserved behavior observed in diverse animal species, which is essential for their survival and food acquisition (Zhao et al., 2023). It encompasses a series of sequential actions including prey search, pursuit, attack, and consumption (Yu H. et al., 2021). PAG plays a vital role in the initiation, execution, and coordination of predatory hunting. Studies have shown that the rostral lateral PAG influences the transition from maternal to hunting behavior in morphine-treated dams, while lesions of the same region impair the ability of mice to effectively chase or attack the prey (Franklin, 2019). Optrode recordings and photoinhibition experiments have further revealed the distinct functions of different cell types within the LPAG. LPAG GABAergic neurons have been found to be critical for prey search, pursuit, and attack, while LPAG glutamate neurons have been found to selectively regulate the attack phase (Yu H. et al., 2021).

With regard to the orchestration of hunting behaviors, single-unit recordings have unveiled that the LPAG neurons can be categorized into seven clusters that encode different predatory actions in a sequential pattern, which align with different hunting actions (Yu H. et al., 2021). This mechanism can ensure accurate and stable execution while providing flexibility and permitting adaptability to environmental changes. Notably, some researchers have proposed that the LPAG acts as a regulatory brake, rather than an accelerator, for predation (Rossier et al., 2021). This hypothesis suggests that the LPAG directly inhibits the neural activity of defensive behaviors, such as risk assessment, fear, and flight, thereby facilitating fearless predatory behavior (Rossier et al., 2021).

PAG receives substantial projections from regions including the CeA, lateral hypothalamus (LH), medial preoptic area (MPA), zona incerta (ZI), and basal forebrain (BF), making it a potential integration center for hunting-related information. Furthermore, it encodes the sequential organization of the aforementioned hunting actions (Han et al., 2017; Park et al., 2018; Zhao et al., 2019; Roman-Ortiz et al., 2021; Rossier et al., 2021; Tan et al., 2022). For example, GABAergic inputs from the CeA mainly control prey pursuit (Han et al., 2017), while those from the LH mainly regulate prey attack (Tan et al., 2022). Additionally, GABAergic inputs from ZI mainly control the introduction and chase phases (Zhao et al., 2019). Table 1 summarizes the major brain nuclei that project to the PAG and their corresponding effects on predatory hunting following optogenetic stimulation.

**TABLE 1:** Diverse functions mediated by PAG-associated neural circuits and related neurotransmitters.

Function	Neural circuits	Neurotransmitter	Related behaviors
Pain regulation	PAG-RVM	Glu; GABA	Pain perception (Samineni et al., 2017; Kuner and Kuner, 2021)
	LPAG/VLPAG-RVM	Somatostatin	The central processing of neuropathic pain (Zhang et al., 2023)
	VLPAG-RVM	Dynorphin/Glu	Responses to cold, thermal, itch, and nociception (McPherson and Ingram, 2022)
	VLPAG/DR*^DA^*-RVM	DA/Glu	Antinociception (Xie et al., 2023)
	VLPAG-DR*^Glu^*-RVM	GABA	Nociception (Xie et al., 2023)
	PAG-RVM-SC	\	Descending analgesia enhancement (Kim et al., 2018)
	PAG-LC-SC	\	Descending analgesia inhibition (Kim et al., 2018)
	VLPAG-CM-BLA	\	Neuropathic pain regulation (Sun et al., 2020)
	PVN-VLPAG-SC	oxytocin	Analgesia in both inflammatory and neuropathic pain models (Iwasaki et al., 2023)
	VLPAG/DR-BST	DA	Inhibition of thermal and mechanical nociception and response to inflammatory injury (Kim et al., 2018; Yu W. et al., 2021)
	ACC-DLPAG/LPAG	Glu	Enhancement of both reflexive and active avoidance behavior toward pain (Lee et al., 2022)
	CeA-PAG	Corticotropin; GABA	Acute stress-related analgesia and pain modulation (Kuner and Kuner, 2021; Mercer Lindsay et al., 2021; McPherson and Ingram, 2022)
	vLGN/IGL-LPAG/VLPAG	\	Antinociceptive effects of bright light treatment (Hu et al., 2022)
	LH-VLPAG	Glu	Pain behaviors (Siemian et al., 2021)
	BLA-mPFC-VLPAG	\	Nociception, pain affect and cognition (Huang et al., 2019; Kuner and Kuner, 2021)
	Sm-VLO-PAG	Opioid peptides/5-HT/DA/Glu/GABA	Antinociception (Huo et al., 2008; Tang et al., 2009)
	MPA-PAG-RVM	\	Analgesia and descending nociception (Loyd and Murphy, 2013)
	VTA-PL mPFC-VLPAG	DA	Antinociception (Huang et al., 2020)
	Medial thalamus-PAG	Glu	Pain modulation (Mercer Lindsay et al., 2021)
Defensive behavior	CeA-VLPAG	GABA	Conditioned freezing (Tovote et al., 2016; Reis et al., 2023)
	ACC-DLPAG/LPAG	\	Defensive behaviors (Reis et al., 2023)
	VMH-DLPAG	Glu	Freezing and immobility (Wang et al., 2015)
	PMD-DLPAG	Glu/Cholecystokinin	Escape vigor (Wang et al., 2021a)
	AHN-VLPAG	GABA	Defensive attacks and biting (Xie et al., 2022)
	LH-LPAG/VLPAG	Glu	Evasion and unconditioned aversion (Li Y. et al., 2018)
	DR/MR-DLPAG/LPAG/VLPAG	\	Escape and avoidance (Reis et al., 2023)
	ZI-LPAG	GABA	Investigatory behavior (Ahmadlou et al., 2021)
	SN-VLPAG	GABA	Exploratory locomotion (Kirouac et al., 2004)
	PL/IL-VLPAG	\	Aversion and fear generalization (Reis et al., 2023)
	mSC-DPAG	Glu	The initiation of unconditioned escape (Evans et al., 2018)
	FN-VLPAG	Glu	Learned defensive responses (da Silva et al., 2023)
	VTA*^DA^*-mPFC-DPAG	DA	Place avoidance and defensive behaviors (Vander Weele et al., 2018)
	VLPAG-Mc	Glu	Freezing behaviors (Tovote et al., 2016)
	VLPAG-VTA	GABA	Freezing behaviors (St Laurent et al., 2020)
	mCbN-VLPAG	Glu	Freezing behaviors (Vaaga et al., 2020)
Predatory hunting	CeA-LPAG/VLPAG	GABA	Locomotion during prey pursuit (Zhao et al., 2023)
	LH-LPAG/VLPAG	Glu; GABA	Evasion; Predatory hunting (Rossier et al., 2021; Zhao et al., 2023)
	MPA-LH-VLPAG	CaMKIIα	Predatory eating in hunting behavior (Tan et al., 2022)
	MPA-VLPAG	CaMKIIα	Predation (Tan et al., 2022)
	ZI-PAG	GABA	Hunting behavior (Zhao et al., 2019)
	BF-PAG	GABA	Hunting and instrumental responding and consumption for food (Roman-Ortiz et al., 2021)
	BST-VLPAG	GABA	Feeding behavior (Hao et al., 2019)
Anxiety and depression	MRN-DPAG	5-HT	Anxiety (Vázquez-León et al., 2023)
	dmPFC-VLPAG	Glu	Maintenance of pain thresholds and antianxiety behaviors under normal conditions (Yin et al., 2020)
	PL mPFC-PAG	Glu	Motivation and depression-related behaviors (Silva and McNaughton, 2019)
	MPA-PAG	Glu	Anxiety-like behaviors (Zhang et al., 2021)
Cardiovascular and respiratory regulation	PAG-RPa	\	Cardiac output during defense (Moraes et al., 2020)
	POA-VLPAG-RN	Adrenaline	Hypotension caused by LPS (Millington et al., 2016)
	mPFC-VLPAG-RVLM	Glu	Increase of tachycardia (Alikhani et al., 2021)
	POA-DPAG	Adrenaline	Hypotension caused by LPS (Mirzaii-Dizgah et al., 2022)
	VLPAG-CVLM*^GABA^*-RVLM	Glu	Decrease of vasodilator nerve acticity in RVLM (Alikhani et al., 2021)
	VLPAG-CMM^5–HT^-RVLM	Glu	De-inhibition of pressor response to RVLM (Alikhani et al., 2021)
	VLPAG-NTS	NMDA	Hemorrhagic hypotension reverse and increase of HR (Barbosa et al., 2017)
	DMH-LPAG/DLPAG^NMDA/5–HT^	\	Increase of HR and mean arterial pressure; attenuation of DMH-induced tachycardia (de Menezes et al., 2009; Villela et al., 2009)
	PAG-NRA	\	Respiratory regulation and the articulation related to emotion (Holstege and Subramanian, 2016)
	PAG-KFn/KFn-PAG	\	Regulation of upper airway patency (Trevizan-Baú et al., 2021b)
	PAG-pre-Bötzinger complex	\	Breathing behavior (Ghorbani et al., 2023)
	PVN/PFA/DMH-PAG	\	Respiratory regulation (Fukushi et al., 2019)
	LPAG-VLM ^NK1R^	Glu	Respiratory control (Oka et al., 2012)
Sleep-wake states modulation	VLPAG-SLD^Glu^/LDT^Ach^/LC^NE^	GABA	Decrease of REM sleep (Weber et al., 2018)
	VLPAG-DR^5–HT^/LC^NE^/ VLPAG^GABA^	GABA	Initiation and maintenance of REM sleep (Luppi et al., 2012)
	VM^GABA^/LH^MCH^/POA-VLPAG	GABA/MCH	Promotion of REM sleep (Weber et al., 2015; Kroeger et al., 2019)
	LC-VLPAG^DA^	\	Increase of sleep latency (Porter-Stransky et al., 2019)
	VLPAG/LPAG-caudal medulla oblongata^GABA^	Glu	Promotion of NREM sleep (Kashiwagi et al., 2020)
Others	PAG-NTS	GABA	Inhibition of cough and respiratory sensory management (Zhong et al., 2019; Chen et al., 2022)
	VLPAG/LPAG-PMC	\	Initiation of micturition response (Rao et al., 2022)
	DLPAG-PMC	GABA	Voiding inhibition (Zare et al., 2019)
	DLPAG-VLPAG	GABA	Inhibition of micturition reflex (Numata et al., 2008)
	LBP-L/VLPAG	Glu	Modulation in itching sensation (Li et al., 2021)

The symbol “/” means to summarize nucleus or neurotransmitters which mediate the same function. The symbol “;” means to distinguish different functions and its relative neurotransmitters. The symbol “\” means the neurotransmitter is unknown.

### 3.4 Involvement of PAG in negative emotions of anxiety and depression

In response to uncontrollable stressors, such as pain and threatening stimuli described in the previous text, animals may exhibit negative emotions such as anxiety and depression. These emotions are closely intertwined with the modulation of threat and pain. For instance, anxiety is crucial for alerting and preparing individuals to cope with environmental threats, thereby promoting survival when appropriately regulated (Lowery-Gionta et al., 2018). However, excessive or persistent anxiety can disrupt daily functioning and potentially lead to mental health disorders. Moreover, chronic pain can induce anxiety and depression, while negative emotions can further worsen chronic pain (Yin et al., 2020). Depression and chronic pain are frequently comorbid; up to 85% of patients with chronic pain experience depression (Sheng et al., 2017). Therefore, there is a complex interplay between negative emotions, defensive behaviors, and pain modulation, where each component can influence and interact with the others.

#### 3.4.1 Role of PAG in anxiety

Anxiety refers to the emotional and behavioral responses triggered by potential threats, involving autonomic arousal and increased avoidance behavior (Lowery-Gionta et al., 2018; Vázquez-León et al., 2023). PAG is widely recognized as being involved in anxiety processes. Some investigators believe that the DLPAG is a local conflict resolution center where immediate appetitive and aversive input converge, suggesting that it may be involved in anxiety phenomena arising from conflicting goals (Silva and McNaughton, 2019). Experimental assessments of anxiety levels in rodents, such as the open field test, elevated plus maze (EPM) test, and elevated T-maze tests, can provide supporting evidence (Bertoglio et al., 2005; Pobbe et al., 2011; Zangrossi and Graeff, 2014; Vázquez-León et al., 2023). In the EPM test, rodents exhibit a phenomenon known as one-trial tolerance, where they no longer respond to anxiolytic-like drugs during retesting (Trial 2) after experiencing EPM in Trial 1 (Bertoglio et al., 2005). However, after blocking the DLPAG activity, the anxiolytic effects of systemic benzodiazepine injections were again observed during Trial 2, indicating the involvement of this region in the anxiety-like effects of the one-trial tolerance phenomenon (Bertoglio et al., 2005). Additionally, studies on the VLPAG have demonstrated that functional inhibition of VLPAG GABA neurons leads to enhanced anxiety-like behaviors (Lowery-Gionta et al., 2018), while modulating the dorsal medial prefrontal cortex (dmPFC)-VLPAG pathway may play a role in chronic pain and the emergence of anxiety-like behaviors, as indicated by reduced exploration in the central area in the open field test and the open arms of EPM (Yin et al., 2020).

PAG contains abundant serotonergic neurons, and 5-HT receptors have been implicated in the pathophysiology of anxiety (Pobbe et al., 2011). Intra-DPAG infusion of 5-HT in rats increased inhibitory avoidance acquisition in the elevated T-maze tests, suggesting an anxiogenic effect (Pobbe et al., 2011). The DPAG shows an interaction between 5-HT and NMDA receptors, potentially regulating anxiety-related behaviors (Moraes et al., 2008). Additionally, the PAG is closely linked to the hypothalamic-pituitary-adrenal axis through strong reciprocal 5-HT fibers, which contribute to the modulation of anxiety-like behaviors via serotonergic pathways (Vázquez-León et al., 2023).

#### 3.4.2 Involvement of PAG in depression

The accumulation of stressors heightens the susceptibility to depressive disorders (Peng et al., 2022). PAG is implicated in responding to uncontrollable stress and exhibiting the related behavioral responses. In forced swimming experiments, immobility behaviors resembling depression are observed with activation of the entire PAG region (Lino-de-Oliveira et al., 2006). Mice exhibiting depression-like symptoms show heightened DeltaFosB expression in the VLPAG (Berton et al., 2007). Conversely, treatment with antidepressant drugs reduces immobility behavior and prevents the activation of PAG neurons (Lino-de-Oliveira et al., 2006). Both chronic restraint stress and chronic pain have been shown to diminish glutamatergic neuron activity in the VLPAG, while the latter also weakens the VLPAG-ventral tegmental area (VTA) circuit, which may contribute to the observed co-occurrence of pain and depression (Peng et al., 2022; Lee et al., 2023).

### 3.5 Regulatory effects of PAG on the autonomic nervous system

#### 3.5.1 The involvement of PAG in the regulation of cardiovascular function

PAG plays an essential role in the connection between the cardiovascular and nociceptive systems. Peripheral nociceptive feelings can cause changes in the cardiovascular system, respiration, mood, and behavior. After stimulation of the nociceptors during the abovementioned defensive and aggressive behaviors, pain signals are transmitted to the central nervous system through myelinated and unmyelinated fibers, including several cardiopulmonary regulation areas, where information is integrated, and complex motor and autonomic nervous responses are triggered. In PAG, LPAG, and DLPAG are involved in hypertension and tachycardia after skin and body injury (Chaitoff et al., 2012). VLPAG mediates hemorrhagic hypotension and visceral nociceptive stimulus-mediated hypotension and bradycardia (Cavun et al., 2001, 2004). VLPAG is involved in the initiation of endotoxic hypotension, and VLPAG inactivation can prevent the decrease in arterial pressure caused by severe bleeding and visceral injury (Millington et al., 2016). In previous studies, microinjection of acetylcholine (Ach) into the DPAG did not affect the average arterial pressure and heart rate (HR) of normotensive rats and did not significantly reduce the cardiovascular parameters of DPAG. As a result, some investigators suggested that DPAG did not participate in cardiovascular activities under normal circumstances (Mirzaii-Dizgah et al., 2022).

Nitric oxide (NO) is a major component of PAG-mediated cardiovascular response and an important substance with multiple peripheral and central effects (NejadShahrokhAbadi et al., 2020; Najaftomaraei et al., 2022). It is synthesized by the NO synthase family and has three different subtypes: endothelial, inducible, and neural. The three subtypes of NO in DLPAG play different roles in regulating cardiovascular responses during mechanical, heat-mediated, and cold-mediated nociception (Chaitoff et al., 2012). DLPAG also contains 5-HT and adrenergic receptors, which play a role in reducing the BP. For example, lipopolysaccharide (LPS) produces a hypertensive effect by inhibiting the adrenergic receptors in DPAG. VLPAG contains Ach, norepinephrine (NE), opioid receptors, and glutamate, which cause different BP and HR responses (Lagatta et al., 2016). Microinjection of δ opioid receptor agonists into the VLPAG decreased the arterial pressure, while microinjection of glutamate into the VLPAG increased the cardiovascular responses in rats with normal BP and hemorrhagic hypotension; these effects were mainly mediated by NMDA receptors (Alikhani et al., 2021). In rats with normal BP, microinjection of Ach into the LPAG reduced the BP and increased the HR, whereas rats with hydralazine-mediated hypotension did not exhibit changes in the systolic and mean arterial pressure but had a significantly increased HR (Ghorbani et al., 2023).

PAG plays a key role in the integration of the cardiovascular regulatory network, which is interrelated with the nuclei affecting the cardiovascular system, such as LH, paraventricular hypothalamic nucleus, medial preoptic nucleus, amygdala, prefrontal cortex, and insular cortex. Moreover, PAG projects to all medullary regions that control BP and HR (Dampney, 2018). Studies have shown that the increase in cardiac responses during the defense reaction may depend on the PAG-raphe pallidus (RPa) pathway (Moraes et al., 2020). The hypothalamic preoptic area (POA) is closely related to hypotension caused by LPS, and its mechanism involves activation of POA neurons, which reduces the arterial pressure through the descending pathway from the POA-VLPAG-raphe nucleus (Millington et al., 2016). Inhibition of adrenergic receptors in the POA-DPAG pathway can prevent LPS-induced hypotension (Mirzaii-Dizgah et al., 2022). DPAG is involved in the motor control of blood vessels via its projections to the caudal ventrolateral medulla (CVLM), nucleus tractus solitarius (NTS), and RVLM. For example, DMPAG receives inputs from the ventrolateral medulla and various parts of the SC and sends outputs to the RVLM. DLPAG also has two-way connections with the dorsal medial hypothalamus (DMH), which also has a major projection to the cuneiform nucleus (CnF) (Mohebbati et al., 2020).

#### 3.5.2 Effects of PAG on respiratory regulation

Direct stimulation of different parts of PAG in cats can cause different effects on respiration. DMPAG stimulation can cause slow deep breathing and dyspnea. Conversely, DLPAG stimulation can cause shortness of breath, whereas LPAG and VLPAG stimulation can produce respiratory changes related to vocalization (Subramanian et al., 2008). Microinjection of bicuculline (GABA-A receptor antagonist) into the DPAG produces similar dose-dependent shortness of breath (Hayward et al., 2003). In anesthetized rats, microinjection of the excitatory amino acid D, L-homocysteine into the DLPAG causes a significant increase in renal sympathetic nerve activity and respiratory activity (Dampney et al., 2013). In a previous study of the role of PAG in CO_2_-driven respiration, the destruction of DLPAG, DMPAG, VLPAG, and LPAG by amanita regulated the hypercapnia ventilation response in rats but did not affect the pulmonary ventilation, arterial pressure, HR, or body temperature (Lopes et al., 2012).

PAG is closely related to the brainstem respiratory center. LPAG and VLPAG receive projections from respiratory regulatory centers, such as medulla Bötzinger complex, pre-Bötzinger complex, Kölliker-Fuse nucleus (KFn), LC, dorsal and caudal raphe, lateral and medial parabrachial nucleus, and paratrigeminal nucleus. Furthermore, LPAG and VLPAG project to the forebrain, including the lateral thalamic nucleus, bed nucleus of stria terminalis, and CeA, and the hindbrain, including the pre-Bötzinger complex, KFn, lateral facial nucleus, posterior oblique nucleus, LC, and dorsal and caudal raphe (Oliveira et al., 2021; Trevizan-Baú et al., 2021a,b; Krohn et al., 2023).

### 3.6 Effects of PAG on sleep and wakefulness states

Sleep-wake disorders cause several psychiatric disorders, and VLPAG is known to play an important role in regulating rapid eye movement (REM) sleep (Sastre et al., 1996). Damage to the VLPAG neurons increases REM sleep in cats, rats, and mice (Petitjean et al., 1975; Lu et al., 2006; Kaur et al., 2009). Activation or inhibition of VLPAG GABAergic neurons by photogenetics and chemical genetics decreases or increases REM sleep, respectively (Hayashi et al., 2015; Weber et al., 2015). Studies have shown the presence of REM sleep-promoting (REM-on) neurons and REM sleep-suppressing (REM-off) neurons in the VLPAG. Activation of GABAergic REM-off neurons in the VLPAG inhibits REM sleep and wakefulness and consolidates non-REM (NREM) sleep. Its mechanism involves the inhibition of the sublaterodorsal tegmental nucleus (SLD) glutamatergic neurons, laterodorsal tegmental nucleus (LDT) Ach and LC NE neurons, and other REM-on neurons (Weber et al., 2018). Inhibition of REM-off neurons is the main mechanism underlying an increase in REM sleep. GABA neurons in the VLPAG can inhibit REM-off neurons, such as the DR 5-HT, LC NE, and VLPAG GABA, through their fiber projections, thus initiating and maintaining REM sleep (Luppi et al., 2012). REM-on neurons in the pons, ventral and dorsal medulla, LH, and POA inhibit VLPAG REM-off neurons and promote REM sleep. For example, the ventral medulla (VM) GABA neurons directly innervate the VLPAG GABA neurons to rapidly initiate REM sleep and prolong its duration (Lu et al., 2006; Weber et al., 2015, 2018). The melanin-concentrating hormone (MCH) neurons in the LH inhibit the VLPAG neurons. This experiment demonstrates for the first time that VLPAG is an important relay station for MCH neurons to promote REM sleep (Kroeger et al., 2019).

Previous studies have shown that the activity of DA neurons in the VLPAG varies with awakening. LC inputs and activation of the VLPAG α1-adrenergic receptor and VLPAG DA neurons increase sleep latency (Porter-Stransky et al., 2019). VLPAG DA neurons are also involved in the regulation of propofol and isoflurane anesthesia in rats, which is mediated by the activation of GABA receptors (Li J. et al., 2018; Liu et al., 2020). Activation of glutamatergic neurons expressing neurotensin in VLPAG/LPAG can significantly promote NREM sleep, and its neural pathway stimulates GABA neurons in caudal medulla oblongata to play a role (Zhong et al., 2019; Kashiwagi et al., 2020). The regulation of sleep-wake in other subregions of PAG is still poorly understood and needs further study.

The dysfunction of PAG is also closely associated with sleep disorders including REM sleep behavior disorder (RBD) and narcolepsy. Research has found PAG abnormalities in those with idiopathic RBD. Noted neurodegeneration has also been observed in the PAG of a patient with RBD and cognitive decline (Iranzo, 2018). Additionally, stimulating the PAG can provoke escape and aggressive behaviors that resemble RBD symptoms, further highlighting its critical role in the manifestation of RBD (Iranzo, 2018). When REM-off neurons in VLPAG weaken, this can lead to a premature switch to REM sleep. This is a key feature of narcolepsy, where the boundaries between wakefulness and REM sleep become blurred and can be predicted by the flip-flop switch model (Lu et al., 2006). In this model, VLPAG GABAergic neurons inhibit REM-on neurons, while its glutamatergic neurons further modulate REM sleep negatively, highlighting the VLPAG’s critical role in managing sleep-wake transitions (Wang et al., 2021c). Narcolepsy’s symptoms are primarily due to insufficient inhibition on REM-off VLPAG/dorsal part of the deep mesencephalic nucleus GABAergic and the waking aminergic neurons (Luppi et al., 2011). This understanding reinforces the significance of targeting PAG functionality in developing treatments for related sleep disorders.

### 3.7 PAG is involved in vocalization and bladder control behavior

PAG plays a key role in vocalization and bladder control and is essential for the survival and development of both humans and animals. It is involved in verbal communication and expression of emotions, urine storage, and urination. The control of PAG over animal sound production, particularly the stimulation of laughter, was demonstrated in chimpanzees in 1915. Stimulation of different PAG regions produces different sounds in a variety of animals. Damage to the PAG area causes irreversible loss of sound production. Animals generally make two types of sounds. The first one is related to communication, which is used by animals to voluntarily transmit information to identify groups (Ruat et al., 2022). The other is related to emotions such as joy, anger, and fear. The PAG region is involved in the regulation of both sound types (Behbehani, 1995). The PAG region serves as a vocal transit station and is mainly divided into three types: excitatory input from the emotion-related pathways from the basal temporal lobe, frontal lobe, limbic system, and basal ganglia; visual, auditory, taste, and somatosensory information provided to the PAG by the superior and inferior colliculi, nucleus of the solitary tract, spinal nucleus of the trigeminal nerve, and dorsal horn of the SC; and projections of the facial region of the motor cortex. PAG integrates the received information and produces the corresponding sound (Jürgens, 1994; Klingbeil et al., 2021; Subramanian et al., 2021). Conversely, PAG has a strong projection to the caudal medulla, which is related to certain emotional expressions.

For bladder control behavior, four subregions of VLPAG are involved. Electrical and chemical stimulation causes bladder contraction and increases the BP in cats (Taniguchi et al., 2002). Microinjection of D, L-homocysteine into the VLPAG region reduced the frequency of urination but did not disrupt the coordinated pattern of urination (Stone et al., 2015). In a previous study, rats were electrically stimulated by implantation of bipolar stimulation electrodes into the bladder wall, and the glutaminergic neurons that mainly activated VLPAG were identified by staining of brain tissue (Zare et al., 2018). A significant increase in c-Fos expression in the VLPAG was observed after stress urinary incontinence was induced by transurethral dissolution in rats (Ko et al., 2010). PAG has significant connections with the cortex (prefrontal lobe, cingulate gyrus, and insular gyrus), diencephalon (MPA of thalamus and hypothalamus), pontine micturition center (PMC), and SC (sacral ganglion), which are involved in the initiation of the micturition reflex (Zare et al., 2019; Rao et al., 2022).

## 4 Potential clinical significance and treatment implications of PAG dysfunction in pain, cardiovascular disorders, and psychiatric disorders

Deep brain stimulation, which involves electrical stimulation of the brain parenchyma through implanted electrodes, is a neuroregulatory therapy. PAG is the most extensively studied brainstem target for deep brain stimulation. It is used as a treatment of pain, including neuropathic pain, that is difficult to treat with drugs. PAG-deep brain stimulation also regulates various autonomic nervous system functions. For example, the BP of a hypertensive patient decreased after PAG stimulation for chronic pain. Stimulation of ventral PAG is beneficial for refractory hypertension, while stimulation of DPAG may improve orthostatic hypotension (Pereira et al., 2010; Patel et al., 2011). Furthermore, PAG stimulation increases the maximum bladder capacity (Green et al., 2012). Stimulation of the PAG subdomain in the human body can cause similar changes in BP and HR, which is consistent with the observations in experimental animals. A hypertensive patient who did not achieve adequate BP control with various drugs demonstrated a marked decrease in BP after 6 months of ventral PAG stimulation (Farrell et al., 2019). Furthermore, PAG is essential for driving changes in acute harmful stimulation behavior. High-resolution (7 Tesla) functional magnetic resonance imaging (fMRI) scans of 16 healthy subjects (including 7 females) were performed to evaluate the potential activation of the facial hypothalamus, amygdala, and PAG. The results showed that the signal intensity of LPAG was increased during nociceptive stimulation, while the signal of the other two regions was decreased (Robertson et al., 2022).

Chronic cough is a common and refractory symptom in respiratory diseases, and L/VLPAG sends GABAergic fibers to NTS to suppress the urge to cough (Chen et al., 2022). Dyspnea is considered to be the most direct and strongest threat to survival and often causes serious anxiety. The functional division and connection of human PAG have potential clinical significance for certain patients with chronic lung disease and panic disorder (Faull and Pattinson, 2017). Human fMRI studies have shown that PAG is the main target for the diagnosis, prevention, and treatment of neuropsychiatric diseases (George et al., 2019). In total, 48 patients with trauma exposure underwent resting-state functional connectivity change scan 2 weeks after trauma, and self-assessment scales were administered after 6 months. The results showed that the PAG connectivity was significantly altered in patients with post-traumatic stress disorder. The increased connectivity of the PAG-prefrontal cortex and PAG-cingulate cortex can predict the symptoms and severity of traumatic stress disorder (Webb et al., 2020). An fMRI scan study found that PAG activation was interrupted by both voluntary and involuntary laughter (Westermann et al., 2022). Human PAG damage leads to absolute silence, and the projection of various limbic system and prefrontal lobe pathways to the PAG determines the tone of speech, which indicates that PAG is an essential nucleus for voice production (Holstege and Subramanian, 2016).

## 5 Summary and prospects

In conclusion, the PAG exhibits diverse functional characteristics and plays a crucial role in the survival and reproduction of mammals. This complex midbrain region serves as a bridge between the forebrain and brainstem, receiving projections from multiple brain areas and integrating information before transmitting it downstream (Benarroch, 2012). The functions of this region include regulation of negative emotions and emotion-related behaviors, such as fear, anxiety, pain, predation, and defense behaviors, as well as corresponding cardiovascular changes. These functional changes mediated by PAG regulation are partly related to sleep-wake regulation. Currently, only the regulation of sleep arousal by the VLPAG has been studied in detail, while the effects of other subregions of PAG on sleep arousal are still unclear. As a result, further studies are needed. Vital neurotransmitters, including GABA, glutamate, opioids, DA, and 5-HT, intricately regulate the mechanisms underlying the effects of PAG, fine-tuning the balance between nociception and emotional states (Nguyen et al., 2023). Table 1 summarizes further details about the afferent and efferent projections of various regions of PAG, their functions, and related behaviors. Nevertheless, a comprehensive understanding of the functions of PAG is lacking due to the interactions among its subregions and the complex effects of neurotransmitters on different subregion receptors. Further comprehensive research is needed to reveal the complex interactions within PAG and to enhance our understanding of this key brain region.

## Author contributions

HZ: Investigation, Methodology, Writing – original draft. ZZ: Investigation, Writing – original draft. W-XM: Investigation, Writing – original draft. L-XK: Investigation, Writing – original draft. P-CY: Investigation, Writing – original draft. L-FB: Investigation, Writing – original draft. JH: Writing – review & editing. Z-LH: Conceptualization, Funding acquisition, Investigation, Writing – review & editing. Y-QW: Conceptualization, Funding acquisition, Investigation, Supervision, Writing – review & editing.

## References

[B1] Ahmadlou M. Houba J. H. W. van Vierbergen J. F. M. Giannouli M. Gimenez G. A. van Weeghel C. (2021). A cell type-specific cortico-subcortical brain circuit for investigatory and novelty-seeking behavior. *Science* 372:eabe9681 10.1126/science.abe9681 33986154

[B2] Alikhani V. Mohebbati R. Hosseini M. Khajavirad A. Shafei M. N. (2021). Role of the glutamatergic system of ventrolateral periaqueductal gray (vlPAG) in the cardiovascular responses in normal and hemorrhagic conditions in rats. *Iran J. Basic Med Sci.* 24 586–594. 10.22038/ijbms.2021.53181.11978 34249259 PMC8244607

[B3] Bandler R. Keay K. A. Floyd N. Price J. (2000). Central circuits mediating patterned autonomic activity during active vs. passive emotional coping. *Brain Res. Bull.* 53 95–104. 10.1016/s0361-9230(00)00313-0 11033213

[B4] Barbosa R. M. Speretta G. F. Dias D. P. M. Ruchaya P. J. Li H. Menani J. V. (2017). Increased expression of macrophage migration inhibitory factor in the nucleus of the solitary tract attenuates renovascular hypertension in rats. *Am. J. Hypertens.* 30 435–443. 10.1093/ajh/hpx001 28158469 PMC5861587

[B5] Behbehani M. M. (1995). Functional characteristics of the midbrain periaqueductal gray. *Prog. Neurobiol.* 46 575–605. 8545545 10.1016/0301-0082(95)00009-k

[B6] Benarroch E. E. (2012). Periaqueductal gray: An interface for behavioral control. *Neurology.* 78 210–217. 22249496 10.1212/WNL.0b013e31823fcdee

[B7] Bertoglio L. J. Anzini C. Lino-de-Oliveira C. Carobrez A. P. (2005). Enhanced dorsolateral periaqueductal gray activity counteracts the anxiolytic response to midazolam on the elevated plus-maze Trial 2 in rats. *Behav. Brain Res.* 162 99–107. 10.1016/j.bbr.2005.03.010 15922070

[B8] Berton O. Covington H. E. Ebner K. Tsankova N. M. Carle T. L. Ulery P. (2007). Induction of ΔFosB in the periaqueductal gray by stress promotes active coping responses. *Neuron* 55 289–300. 10.1016/j.neuron.2007.06.033 17640529

[B9] Borelli K. G. Ferreira-Netto C. Coimbra N. C. Brandao M. L. (2005). Fos-like immunoreactivity in the brain associated with freezing or escape induced by inhibition of either glutamic acid decarboxylase or GABAA receptors in the dorsal periaqueductal gray. *Brain Res.* 1051 100–111. 15996642 10.1016/j.brainres.2005.05.068

[B10] Bouchet C. A. Ingram S. L. (2020). Cannabinoids in the descending pain modulatory circuit: Role in inflammation. *Pharmacol. Ther.* 209:107495. 10.1016/j.pharmthera.2020.107495 32004514 PMC7183429

[B11] Cavun S. Goktalay G. Millington W. R. (2004). The hypotension evoked by visceral nociception is mediated by delta opioid receptors in the periaqueductal gray. *Brain Res.* 1019 237–245. 10.1016/j.brainres.2004.06.003 15306258

[B12] Cavun S. Resch G. E. Evec A. D. Rapacon-Baker M. M. Millington W. R. (2001). Blockade of delta opioid receptors in the ventrolateral periaqueductal gray region inhibits the fall in arterial pressure evoked by hemorrhage. *J. Pharmacol. Exp. Ther.* 297 612–619. 11303050

[B13] Chaitoff K. A. Toner F. Tedesco A. Maher T. J. Ally A. (2012). Effects of inducible nitric oxide synthase blockade within the periaqueductal gray on cardiovascular responses during mechanical, heat, and cold nociception. *Neurol Sci.* 33 69–78. 10.1007/s10072-011-0661-x 21710130

[B14] Chen Z. Lin M. T. Zhan C. Zhong N. S. Mu D. Lai K. F. (2022). A descending pathway emanating from the periaqueductal gray mediates the development of cough-like hypersensitivity. *iScience* 25:103641. 10.1016/j.isci.2021.103641 35028531 PMC8741493

[B15] Chung G. Shim H. G. Kim C. Y. Ryu H. H. Jang D. C. Kim S. H. (2020). Persistent activity of metabotropic glutamate receptor 5 in the periaqueductal gray constrains emergence of chronic neuropathic pain. *Curr. Biol.* 30 4631–4642.e6. 10.1016/j.cub.2020.09.008 32976802

[B16] da Silva G. N. Seiffert N. Tovote P. (2023). Cerebellar contribution to the regulation of defensive states. *Front. Syst. Neurosci.* 17:1160083. 10.3389/fnsys.2023.1160083 37064160 PMC10102664

[B17] Dampney R. (2018). Emotion and the cardiovascular system: Postulated role of inputs from the medial prefrontal cortex to the dorsolateral periaqueductal gray. *Front. Neurosci.* 12:343. 10.3389/fnins.2018.00343 29881334 PMC5976784

[B18] Dampney R. A. Furlong T. M. Horiuchi J. Iigaya K. (2013). Role of dorsolateral periaqueductal grey in the coordinated regulation of cardiovascular and respiratory function. *Auton. Neurosci.* 175 17–25. 10.1016/j.autneu.2012.12.008 23336968

[B19] de Menezes R. C. Zaretsky D. V. Fontes M. A. DiMicco J. A. (2009). Cardiovascular and thermal responses evoked from the periaqueductal grey require neuronal activity in the hypothalamus. *J. Physiol.* 587 1201–1215. 10.1113/jphysiol.2008.161463 19171660 PMC2674992

[B20] Deng H. Xiao X. Wang Z. (2016). Periaqueductal gray neuronal activities underlie different aspects of defensive behaviors. *J. Neurosci.* 36 7580–7588. 27445137 10.1523/JNEUROSCI.4425-15.2016 PMC6705556

[B21] Drake R. A. Leith J. L. Almahasneh F. Martindale J. Wilson A. W. Lumb B. (2016). Periaqueductal grey EP3 receptors facilitate spinal nociception in arthritic secondary hypersensitivity. *J. Neurosci.* 36 9026–9040. 10.1523/JNEUROSCI.4393-15.2016 27581447 PMC5005717

[B22] Evans D. A. Stempel A. V. Vale R. Ruehle S. Lefler Y. Branco T. (2018). A synaptic threshold mechanism for computing escape decisions. *Nature* 558 590–594. 10.1038/s41586-018-0244-6 29925954 PMC6235113

[B23] Farrell S. M. Green A. Aziz T. (2019). The use of neuromodulation for symptom management. *Brain Sci.* 9:232. 10.3390/brainsci9090232 PMC6769574 31547392

[B24] Faull O. K. Pattinson K. T. S. (2017). The cortical connectivity of the periaqueductal gray and the conditioned response to the threat of breathlessness. *eLife* 6:e21749. 10.7554/eLife.21749 28211789 PMC5332157

[B25] Faull O. K. Subramanian H. H. Ezra M. Pattinson K. T. S. (2019). The midbrain periaqueductal gray as an integrative and interoceptive neural structure for breathing. *Neurosci. Biobehav. Rev.* 98 135–144. 10.1016/j.neubiorev.2018.12.020 30611797

[B26] Franklin T. B. (2019). Recent advancements surrounding the role of the periaqueductal gray in predators and prey. *Front. Behav. Neurosci.* 13:60. 10.3389/fnbeh.2019.00060 31133827 PMC6524621

[B27] Fukushi I. Yokota S. Okada Y. (2019). The role of the hypothalamus in modulation of respiration. *Respir. Physiol. Neurobiol.* 265 172–179. 30009993 10.1016/j.resp.2018.07.003

[B28] George D. T. Ameli R. Koob G. F. (2019). Periaqueductal gray sheds light on dark areas of psychopathology. *Trends Neurosci.* 42 349–360. 10.1016/j.tins.2019.03.004 30955857

[B29] Ghorbani A. Mohebbati R. Rahimi A. Alikhani V. Shafei M. N. (2023). Effect of the cholinergic system of the lateral periaqueductal gray (lPAG) on blood pressure and heart rate in normal and hydralazine hypotensive rats. *Iran J. Basic Med. Sci.* 26 891–898. 10.22038/IJBMS.2023.66838.14660 37427334 PMC10329252

[B30] Green A. L. Stone E. Sitsapesan H. Turney B. W. Coote J. H. Aziz T. Z. (2012). Switching off micturition using deep brain stimulation at midbrain sites. *Ann. Neurol.* 72 144–147. 10.1002/ana.23571 22829274

[B31] Hadjipavlou G. Dunckley P. Behrens T. E. Tracey I. (2006). Determining anatomical connectivities between cortical and brainstem pain processing regions in humans: A diffusion tensor imaging study in healthy controls. *Pain* 123 169–178. 10.1016/j.pain.2006.02.027 16616418

[B32] Han W. Tellez L. A. Rangel M. J. Jr. Motta S. C. Zhang X. Perez I. O. (2017). Integrated control of predatory hunting by the central nucleus of the amygdala. *Cell* 168 311–324.e18. 28086095 10.1016/j.cell.2016.12.027 PMC5278763

[B33] Hao S. Yang H. Wang X. He Y. Xu H. Wu X. (2019). The lateral hypothalamic and BNST GABAergic projections to the anterior ventrolateral periaqueductal gray regulate feeding. *Cell Rep.* 28 616–624.e5. 10.1016/j.celrep.2019.06.051 31315042

[B34] Hayashi Y. Kashiwagi M. Yasuda K. Ando R. Kanuka M. Sakai K. (2015). Cells of a common developmental origin regulate REM/non-REM sleep and wakefulness in mice. *Science* 350 957–961. 10.1126/science.aad1023 26494173

[B35] Hayward L. F. Swartz C. L. Davenport P. W. (2003). Respiratory response to activation or disinhibition of the dorsal periaqueductal gray in rats. *J. Appl. Physiol.* 94 913–922. 12571126 10.1152/japplphysiol.00740.2002

[B36] Holstege G. Subramanian H. H. (2016). Two different motor systems are needed to generate human speech. *J. Comp. Neurol.* 524 1558–1577. 26355872 10.1002/cne.23898

[B37] Hu Z. Mu Y. Huang L. Hu Y. Chen Z. Yang Y. (2022). A visual circuit related to the periaqueductal gray area for the antinociceptive effects of bright light treatment. *Neuron* 110 1712–1727.e7. 10.1016/j.neuron.2022.02.009 35263618

[B38] Huang J. Gadotti V. M. Chen L. Souza I. A. Huang S. Wang D. (2019). A neuronal circuit for activating descending modulation of neuropathic pain. *Nat. Neurosci.* 22 1659–1668. 31501573 10.1038/s41593-019-0481-5

[B39] Huang S. Zhang Z. Gambeta E. Xu S. C. Thomas C. Godfrey N. (2020). Dopamine inputs from the ventral tegmental area into the medial prefrontal cortex modulate neuropathic pain-associated behaviors in mice. *Cell Rep.* 31:107812. 10.1016/j.celrep.2020.107812 32579938

[B40] Huo F. Q. Qu C. L. Li Y. Q. Tang J. S. Jia H. (2008). GABAergic modulation is involved in the ventrolateral orbital cortex 5-HT 1A receptor activation-induced antinociception in the rat. *Pain* 139 398–405. 10.1016/j.pain.2008.05.013 18579305

[B41] Iranzo A. (2018). The REM sleep circuit and how its impairment leads to REM sleep behavior disorder. *Cell Tissue Res.* 373 245–266. 29846796 10.1007/s00441-018-2852-8

[B42] Isosaka T. Matsuo T. Yamaguchi T. Funabiki K. Nakanishi S. Kobayakawa R. (2015). Htr2a-expressing cells in the central amygdala control the hierarchy between innate and learned fear. *Cell* 163 1153–1164. 10.1016/j.cell.2015.10.047 26590419

[B43] Iwasaki M. Lefevre A. Althammer F. Clauss Creusot E. Łąpieś O. Petitjean H. (2023). An analgesic pathway from parvocellular oxytocin neurons to the periaqueductal gray in rats. *Nat. Commun.* 14:1066. 10.1038/s41467-023-36641-7 36828816 PMC9958129

[B44] Jürgens U. (1994). The role of the periaqueductal grey in vocal behaviour. *Behav. Brain Res.* 62 107–117. 7945960 10.1016/0166-4328(94)90017-5

[B45] Kashiwagi M. Kanuka M. Tatsuzawa C. Suzuki H. Morita M. Tanaka K. (2020). Widely distributed neurotensinergic neurons in the brainstem regulate NREM sleep in mice. *Curr. Biol.* 30 1002–1010.e4. 10.1016/j.cub.2020.01.047 32032507

[B46] Kaur S. Thankachan S. Begum S. Liu M. Blanco-Centurion C. Shiromani P. J. (2009). Hypocretin-2 saporin lesions of the ventrolateral periaquaductal gray (vlPAG) increase REM sleep in hypocretin knockout mice. *PLoS One* 4:e6346. 10.1371/journal.pone.0006346 19623260 PMC2709920

[B47] Keay K. A. Bandler R. (2001). Parallel circuits mediating distinct emotional coping reactions to different types of stress. *Neurosci. Biobehav. Rev.* 25 669–678. 10.1016/s0149-7634(01)00049-5 11801292

[B48] Kim J. H. Gangadharan G. Byun J. Choi E. J. Lee C. J. Shin H. S. (2018). Yin-and-yang bifurcation of opioidergic circuits for descending analgesia at the midbrain of the mouse. *Proc. Natl. Acad. Sci. U. S. A.* 115 11078–11083. 30297409 10.1073/pnas.1806082115 PMC6205495

[B49] Kirouac G. J. Li S. Mabrouk G. (2004). GABAergic projection from the ventral tegmental area and substantia nigra to the periaqueductal gray region and the dorsal raphe nucleus. *J. Comp. Neurol.* 469 170–184. 10.1002/cne.11005 14694532

[B50] Klingbeil J. Wawrzyniak M. Stockert A. Brandt M. L. Schneider H. R. Metelmann M. (2021). Pathological laughter and crying: Insights from lesion network-symptom-mapping. *Brain* 144 3264–3276. 10.1093/brain/awab224 34142117

[B51] Ko I. G. Kim S. E. Kim C. J. Jung J. H. Lee S. J. Kim D. H. (2010). Effect of treadmill exercise on leak-point pressure and neuronal activation in brain of rats with stress urinary incontinence. *Int. Neurourol. J.* 14 141–148. 10.5213/inj.2010.14.3.141 21179331 PMC2998400

[B52] Kroeger D. Bandaru S. S. Madara J. C. Vetrivelan R. (2019). Ventrolateral periaqueductal gray mediates rapid eye movement sleep regulation by melanin-concentrating hormone neurons. *Neuroscience* 406 314–324. 10.1016/j.neuroscience.2019.03.020 30890480 PMC6545592

[B53] Krohn F. Novello M. van der Giessen R. S. De Zeeuw C. I. Pel J. J. M. Bosman L. W. J. (2023). The integrated brain network that controls respiration. *Elife* 12:e83654 10.7554/eLife.83654 PMC9995121 36884287

[B54] Kuner R. Kuner T. (2021). Cellular circuits in the brain and their modulation in acute and chronic pain. *Physiol. Rev.* 101 213–258. 32525759 10.1152/physrev.00040.2019

[B55] Lagatta D. C. Ferreira-Junior N. C. Deolindo M. Corrêa F. M. Resstel L. B. (2016). Ventrolateral periaqueductal grey matter neurotransmission modulates cardiac baroreflex activity. *Eur. J. Neurosci.* 44 2877–2884. 10.1111/ejn.13407 27646556

[B56] Lee J. Y. You T. Lee C. H. Im G. H. Seo H. Woo C. W. (2022). Role of anterior cingulate cortex inputs to periaqueductal gray for pain avoidance. *Curr. Biol.* 32 2834–2847.e5. 10.1016/j.cub.2022.04.090 35609604

[B57] Lee M. T. Peng W. H. Wu C. C. Kan H. W. Wang D. W. Teng Y. N. (2023). Impaired ventrolateral periaqueductal gray-ventral tegmental area pathway contributes to chronic pain-induced depression-like behavior in mice. *Mol. Neurobiol.* 60 5708–5724 10.1007/s12035-023-03439-z 37338803

[B58] Li J. Yu T. Shi F. Zhang Y. Duan Z. Fu B. (2018). Involvement of ventral periaqueductal gray dopaminergic neurons in propofol anesthesia. *Neurochem. Res.* 43 838–847. 10.1007/s11064-018-2486-y 29417470

[B59] Li J. N. Ren J. H. He C. B. Zhao W. J. Li H. Dong Y. L. (2021). Projections from the lateral parabrachial nucleus to the lateral and ventral lateral periaqueductal gray subregions mediate the itching sensation. *Pain* 162 1848–1863. 10.1097/j.pain.0000000000002193 33449512

[B60] Li Y. Zeng J. Zhang J. Yue C. Zhong W. Liu Z. (2018). Hypothalamic circuits for predation and evasion. *Neuron* 97 911–924.e5. 10.1016/j.neuron.2018.01.005 29398361

[B61] Linnman C. Beucke J.-C. Jensen K. B. Gollub R. L. Kong J. (2012). Sex similarities and differences in pain-related periaqueductal gray connectivity. *Pain* 153 444–454. 10.1016/j.pain.2011.11.006 22154332 PMC3558685

[B62] Lino-de-Oliveira C. de Oliveira R. M. Padua Carobrez A. de Lima T. C. del Bel E. A. Guimaraes F. S. (2006). Antidepressant treatment reduces Fos-like immunoreactivity induced by swim stress in different columns of the periaqueductal gray matter. *Brain Res. Bull.* 70 414–421. 10.1016/j.brainresbull.2006.07.007 17027777

[B63] Liu C. Zhou X. Zhu Q. Fu B. Cao S. Zhang Y. (2020). Dopamine neurons in the ventral periaqueductal gray modulate isoflurane anesthesia in rats. *CNS Neurosci. Ther.* 26 1121–1133. 10.1111/cns.13447 32881314 PMC7564192

[B64] Liu D. Li S. Ren L. Liu X. Li X. Wang Z. (2022). Different coding characteristics between flight and freezing in dorsal periaqueductal gray of mice during exposure to innate threats. *Anim. Model Exp. Med.* 5 491–501. 10.1002/ame2.12276 36225094 PMC9773308

[B65] Lopes L. T. Patrone L. G. Bícego K. C. Coimbra N. C. Gargaglioni L. H. (2012). Periaqueductal gray matter modulates the hypercapnic ventilatory response. *Pflugers Arch.* 464 155–166. 10.1007/s00424-012-1119-6 22665049

[B66] Lopez-Canul M. Palazzo E. Dominguez-Lopez S. Luongo L. Lacoste B. Comai S. (2015). Selective melatonin MT2 receptor ligands relieve neuropathic pain through modulation of brainstem descending antinociceptive pathways. *Pain* 156 305–317. 10.1097/01.j.pain.0000460311.71572.5f 25599452

[B67] Lowery-Gionta E. G. DiBerto J. Mazzone C. M. Kash T. L. (2018). GABA neurons of the ventral periaqueductal gray area modulate behaviors associated with anxiety and conditioned fear. *Brain Struct. Funct.* 223 3787–3799. 10.1007/s00429-018-1724-z 30076467

[B68] Loyd D. R. Morgan M. M. Murphy A. Z. (2007). Morphine preferentially activates the periaqueductal gray–rostral ventromedial medullary pathway in the male rat: A potential mechanism for sex differences in antinociception. *Neuroscience* 147 456–468. 10.1016/j.neuroscience.2007.03.053 17540508 PMC1949345

[B69] Loyd D. R. Murphy A. Z. (2013). “Forebrain modulation of the periaqueductal gray and its role in pain,” in *Encyclopedia of Pain*, eds Gebhart G. F. Schmidt R. F. (Berlin: Springer), 1297–1303.

[B70] Lu J. Sherman D. Devor M. Saper C. B. (2006). A putative flip-flop switch for control of REM sleep. *Nature* 441 589–594. 16688184 10.1038/nature04767

[B71] Lumb B. M. (2004). Hypothalamic and midbrain circuitry that distinguishes between escapable and inescapable pain. *News Physiol. Sci.* 19 22–26. 10.1152/nips.01467.2003 14739399

[B72] Luppi P. H. Clément O. Sapin E. Gervasoni D. Peyron C. Léger L. (2011). The neuronal network responsible for paradoxical sleep and its dysfunctions causing narcolepsy and rapid eye movement (REM) behavior disorder. *Sleep Med. Rev.* 15 153–163. 21115377 10.1016/j.smrv.2010.08.002

[B73] Luppi P. H. Clement O. Sapin E. Peyron C. Gervasoni D. Léger L. (2012). Brainstem mechanisms of paradoxical (REM) sleep generation. *Pflugers Arch.* 463 43–52. 22083642 10.1007/s00424-011-1054-y

[B74] Ma W. X. Li L. Kong L. X. Zhang H. Yuan P. C. Huang Z. L. (2023). Whole-brain monosynaptic inputs to lateral periaqueductal gray glutamatergic neurons in mice. *CNS Neurosci. Ther.* 29 4147–4159 37424163 10.1111/cns.14338 PMC10651995

[B75] Magoun H. W. Atlas D. Ingersoll E. H. Ranson S. W. (1937). Associated facial, vocal and respiratory components of emotional expression: An experimental study. *J. Neurol Psychopathol.* 17 241–255. 21623397 10.1136/jnnp.s1-17.67.241 PMC1039149

[B76] McPherson K. B. Ingram S. L. (2022). Cellular and circuit diversity determines the impact of endogenous opioids in the descending pain modulatory pathway. *Front. Syst. Neurosci.* 16:963812. 10.3389/fnsys.2022.963812 36045708 PMC9421147

[B77] Melzack R. Stotler W. A. Livingston W. K. (1958). Effects of discrete brainstem lesions in cats on perception of noxious stimulation. *J. Neurophysiol.* 21 353–367. 10.1152/jn.1958.21.4.353 13576179

[B78] Mercer Lindsay N. Chen C. Gilam G. Mackey S. Scherrer G. (2021). Brain circuits for pain and its treatment. *Sci. Transl. Med.* 13:eabj7360. 10.1126/scitranslmed.abj7360 PMC8675872 34757810

[B79] Millington W. R. Yilmaz M. S. Feleder C. (2016). The initial fall in arterial pressure evoked by endotoxin is mediated by the ventrolateral periaqueductal gray. *Clin. Exp. Pharmacol. Physiol.* 43 612–615. 10.1111/1440-1681.12573 27009880 PMC4860143

[B80] Mirzaii-Dizgah I. Shafei M. N. Mohebbati R. Alikhani V. (2022). Cardiovascular effect of dorsal periaqueductal gray during lipopolysaccharide-induced hypotension. *Basic Clin. Neurosci.* 13 175–184. 10.32598/bcn.2022.2830.1 36425944 PMC9682318

[B81] Mohebbati R. Hosseini M. Khazaei M. Khajavi Rad A. Shafei M. N. (2020). Involvement of the 5-HT(1A) receptor of the cuneiform nucleus in the regulation of cardiovascular responses during normal and hemorrhagic conditions. *Iran J. Basic Med. Sci.* 23 858–864. 10.22038/ijbms.2020.40453.9579 32774806 PMC7395185

[B82] Mokhtar M. Singh P. (2023). *Neuroanatomy, Periaqueductal Gray.* Treasure Island, FL: StatPearls Publishing. 32119278

[B83] Moraes C. L. Bertoglio L. J. Carobrez A. P. (2008). Interplay between glutamate and serotonin within the dorsal periaqueductal gray modulates anxiety-related behavior of rats exposed to the elevated plus-maze. *Behav. Brain Res.* 194 181–186. 10.1016/j.bbr.2008.07.005 18675851

[B84] Moraes G. C. A. Mendonca M. M. Mourao A. A. Graziani D. Pinto M. C. X. Ferreira P. M. (2020). Ventromedial medullary pathway mediating cardiac responses evoked from periaqueductal gray. *Auton. Neurosci.* 228:102716. 10.1016/j.autneu.2020.102716 32882606

[B85] Morgan M. M. Whittier K. L. Hegarty D. M. Aicher S. A. (2008). Periaqueductal gray neurons project to spinally projecting GABAergic neurons in the rostral ventromedial medulla. *Pain* 140 376–386. 18926635 10.1016/j.pain.2008.09.009 PMC2704017

[B86] Motta S. C. Carobrez A. P. Canteras N. S. (2017). The periaqueductal gray and primal emotional processing critical to influence complex defensive responses, fear learning and reward seeking. *Neurosci. Biobehav. Rev.* 76 39–47. 10.1016/j.neubiorev.2016.10.012 28434586

[B87] Najaftomaraei M. Ghorbani A. Rahimi A. Mohebbati R. Sherkat S. Shafei M. N. (2022). The role of nitric oxide in the dorsomedial periaqueductal gray (dmPAG) column in cardiovascular responses in urethane-anesthetized male rats. *Anim. Model Exp. Med.* 5 557–564. 10.1002/ame2.12292 36415083 PMC9773306

[B88] Nashold B. S. Jr. Wilson W. P. Slaughter D. G. (1969). Sensations evoked by stimulation in the midbrain of man. *J. Neurosurg.* 30 14–24. 4885810 10.3171/jns.1969.30.1.0014

[B89] NejadShahrokhAbadi R. Zangouei A. S. Mohebbati R. Shafei M. N. (2020). Determining the cardiovascular effects of nitric oxide in the dorsolateral Periaqueductal Gray (dlPAG) in anaesthetised rats. *J. Taibah Univ. Med. Sci.* 15 502–508. 33318742 10.1016/j.jtumed.2020.10.004 PMC7715464

[B90] Nguyen E. Grajales-Reyes J. G. Gereau R. W. T. Ross S. E. (2023). Cell type-specific dissection of sensory pathways involved in descending modulation. *Trends Neurosci.* 46 539–550. 10.1016/j.tins.2023.04.002 37164868 PMC10836406

[B91] Numata A. Iwata T. Iuchi H. Taniguchi N. Kita M. Wada N. (2008). Micturition-suppressing region in the periaqueductal gray of the mesencephalon of the cat. *Am. J. Physiol. Regul. Integr. Comp. Physiol.* 294 R1996–R2000. 10.1152/ajpregu.00393.2006 18385467

[B92] Oka T. Yokota S. Tsumori T. Niu J. G. Yasui Y. (2012). Glutamatergic neurons in the lateral periaqueductal gray innervate neurokinin-1 receptor-expressing neurons in the ventrolateral medulla of the rat. *Neurosci. Res.* 74 106–115. 10.1016/j.neures.2012.08.001 22921710

[B93] Oliveira L. M. Takakura A. C. Moreira T. S. (2021). Forebrain and hindbrain projecting-neurons target the post-inspiratory complex cholinergic neurons. *Neuroscience* 476 102–115. 10.1016/j.neuroscience.2021.09.015 34582982

[B94] Park S. G. Jeong Y. C. Kim D. G. Lee M. H. Shin A. Park G. (2018). Medial preoptic circuit induces hunting-like actions to target objects and prey. *Nat. Neurosci.* 21 364–372. 29379117 10.1038/s41593-018-0072-x

[B95] Patel N. K. Javed S. Khan S. Papouchado M. Malizia A. L. Pickering A. E. (2011). Deep brain stimulation relieves refractory hypertension. *Neurology* 76 405–407. 10.1212/WNL.0b013e3182088108 21263142 PMC3034421

[B96] Peng B. Jiao Y. Zhang Y. Li S. Chen S. Xu S. (2023). Bulbospinal nociceptive ON and OFF cells related neural circuits and transmitters. *Front. Pharmacol.* 14:1159753. 10.3389/fphar.2023.1159753 37153792 PMC10157642

[B97] Peng W. H. Kan H. W. Ho Y. C. (2022). Periaqueductal gray is required for controlling chronic stress-induced depression-like behavior. *Biochem. Biophys. Res. Commun.* 593 28–34. 35051779 10.1016/j.bbrc.2022.01.025

[B98] Pereira E. A. Lu G. Wang S. Schweder P. M. Hyam J. A. Stein J. F. (2010). Ventral periaqueductal grey stimulation alters heart rate variability in humans with chronic pain. *Exp. Neurol.* 223 574–581. 10.1016/j.expneurol.2010.02.004 20178783

[B99] Pernía-Andrade A. J. Wenger N. Esposito M. S. Tovote P. (2021). Circuits for state-dependent modulation of locomotion. *Front. Hum. Neurosci.* 15:745689. 10.3389/fnhum.2021.745689 34858153 PMC8631332

[B100] Petitjean F. Sakai K. Blondaux C. Jouvet M. (1975). Hypersomnia by isthmic lesion in cat. II. Neurophysiological and pharmacological study. *Brain Res.* 88 439–453. 10.1016/0006-8993(75)90656-3 166726

[B101] Pobbe R. L. Zangrossi H. Jr. Blanchard D. C. Blanchard R. J. (2011). Involvement of dorsal raphe nucleus and dorsal periaqueductal gray 5-HT receptors in the modulation of mouse defensive behaviors. *Eur. Neuropsychopharmacol.* 21 306–315. 10.1016/j.euroneuro.2010.05.004 20570114 PMC3250220

[B102] Porter-Stransky K. A. Centanni S. W. Karne S. L. Odil L. M. Fekir S. Wong J. C. (2019). Noradrenergic transmission at alpha1-adrenergic receptors in the ventral periaqueductal gray modulates arousal. *Biol. Psychiatry* 85 237–247. 10.1016/j.biopsych.2018.07.027 30269865 PMC6326840

[B103] Rao Y. Gao Z. Li X. Li X. Li J. Liang S. (2022). Ventrolateral periaqueductal gray neurons are active during urination. *Front. Cell Neurosci.* 16:865186. 10.3389/fncel.2022.865186 35813503 PMC9259957

[B104] Reis F. Liu J. Schuette P. J. Lee J. Y. Maesta-Pereira S. Chakerian M. (2021). Shared dorsal periaqueductal gray activation patterns during exposure to innate and conditioned threats. *J. Neurosci.* 41 5399–5420. 10.1523/JNEUROSCI.2450-20.2021 33883203 PMC8221602

[B105] Reis F. Mobbs D. Canteras N. S. Adhikari A. (2023). Orchestration of innate and conditioned defensive actions by the periaqueductal gray. *Neuropharmacology* 228:109458. 10.1016/j.neuropharm.2023.109458 36773777 PMC13041564

[B106] Resstel L. B. M. Lisboa S. F. Aguiar D. C. Corrêa F. M. A. Guimarães F. S. (2008). Activation of CB1 cannabinoid receptors in the dorsolateral periaqueductal gray reduces the expression of contextual fear conditioning in rats. *Psychopharmacology* 198 405–411. 18446325 10.1007/s00213-008-1156-1

[B107] Reynolds D. V. (1969). Surgery in the rat during electrical analgesia induced by focal brain stimulation. *Science* 164 444–445. 4887743 10.1126/science.164.3878.444

[B108] Robertson R. V. Crawford L. S. Meylakh N. Macey P. M. Macefield V. G. Keay K. A. (2022). Regional hypothalamic, amygdala, and midbrain periaqueductal gray matter recruitment during acute pain in awake humans: A 7-Tesla functional magnetic resonance imaging study. *Neuroimage* 259:119408. 10.1016/j.neuroimage.2022.119408 35752415

[B109] Roman-Ortiz C. Guevara J. A. Clem R. L. (2021). GABAergic basal forebrain projections to the periaqueductal gray promote food consumption, reward and predation. *Sci. Rep.* 11:22638. 10.1038/s41598-021-02157-7 34811442 PMC8608827

[B110] Roncon C. M. Yamashita P. S. M. Frias A. T. Audi E. A. Graeff F. G. Coimbra N. C. (2017). μ-Opioid and 5-HT1A receptors in the dorsomedial hypothalamus interact for the regulation of panic-related defensive responses. *J. Psychopharmacol.* 31 715–721. 10.1177/0269881117693747 28583050

[B111] Rossier D. La Franca V. Salemi T. Natale S. Gross C. T. (2021). A neural circuit for competing approach and defense underlying prey capture. *Proc. Natl. Acad. Sci. U. S. A.* 118:e2013411118 10.1073/pnas.2013411118 33876745 PMC8053977

[B112] Ruat J. Genewsky A. J. Heinz D. E. Kaltwasser S. F. Canteras N. S. Czisch M. (2022). Why do mice squeak? Toward a better understanding of defensive vocalization. *iScience* 25:104657. 10.1016/j.isci.2022.104657 35845167 PMC9283514

[B113] Samineni V. K. Grajales-Reyes J. G. Copits B. A. O’Brien D. E. Trigg S. L. Gomez A. M. (2017). Divergent modulation of nociception by glutamatergic and GABAergic neuronal subpopulations in the periaqueductal gray. *eNeuro* 4:ENEURO.0129-16.2017 10.1523/ENEURO.0129-16.2017 28374016 PMC5370278

[B114] Sastre J. P. Buda C. Kitahama K. Jouvet M. (1996). Importance of the ventrolateral region of the periaqueductal gray and adjacent tegmentum in the control of paradoxical sleep as studied by muscimol microinjections in the cat. *Neuroscience* 74 415–426. 10.1016/0306-4522(96)00190-x 8865193

[B115] Schottelkotte K. M. Crone S. A. (2022). Forebrain control of breathing: Anatomy and potential functions. *Front. Neurol.* 13:1041887. 10.3389/fneur.2022.1041887 36388186 PMC9663927

[B116] Sheng J. Liu S. Wang Y. Cui R. Zhang X. (2017). The link between depression and chronic pain: Neural mechanisms in the brain. *Neural Plast.* 2017 1–10. 10.1155/2017/9724371 PMC5494581 28706741

[B117] Siemian J. N. Arenivar M. A. Sarsfield S. Borja C. B. Erbaugh L. J. Eagle A. L. (2021). An excitatory lateral hypothalamic circuit orchestrating pain behaviors in mice. *Elife* 10:e66446 10.7554/eLife.66446 34042586 PMC8159376

[B118] Silva C. McNaughton N. (2019). Are periaqueductal gray and dorsal raphe the foundation of appetitive and aversive control? A comprehensive review. *Progress in neurobiology* 177 33–72. 10.1016/j.pneurobio.2019.02.001 30786258

[B119] St Laurent R. Martinez Damonte V. Tsuda A. C. Kauer J. A. (2020). Periaqueductal gray and rostromedial tegmental inhibitory afferents to VTA have distinct synaptic plasticity and opiate sensitivity. *Neuron* 106 624–636.e4. 10.1016/j.neuron.2020.02.029 32191871 PMC7244388

[B120] Stone E. Coote J. H. Lovick T. A. (2015). Effect of electrical vs. Chemical deep brain stimulation at midbrain sites on micturition in anaesthetized rats. *Acta Physiol.* 214 135–145. 10.1111/apha.12491 25778550

[B121] Subramanian H. H. Balnave R. J. Holstege G. (2008). The midbrain periaqueductal gray control of respiration. *J. Neurosci.* 28 12274–12283. 19020021 10.1523/JNEUROSCI.4168-08.2008 PMC6671706

[B122] Subramanian H. H. Balnave R. J. Holstege G. (2021). Microstimulation in different parts of the periaqueductal gray generates different types of vocalizations in the cat. *J. Voice* 35 804.e9–804.e25. 10.1016/j.jvoice.2020.01.022 32147316

[B123] Sun Y. Wang J. Liang S. H. Ge J. Lu Y. C. Li J. N. (2020). Involvement of the ventrolateral periaqueductal gray matter-central medial thalamic nucleus-basolateral amygdala pathway in neuropathic pain regulation of rats. *Front. Neuroanat.* 14:32. 10.3389/fnana.2020.00032 32792913 PMC7394700

[B124] Tan N. Shi J. Xu L. Zheng Y. Wang X. Lai N. (2022). Lateral hypothalamus calcium/calmodulin-dependent protein kinase II α neurons encode novelty-seeking signals to promote predatory eating. *Research* 2022:9802382. 10.34133/2022/9802382 36061821 PMC9394055

[B125] Tang J. S. Qu C. L. Huo F. Q. (2009). The thalamic nucleus submedius and ventrolateral orbital cortex are involved in nociceptive modulation: A novel pain modulation pathway. *Prog. Neurobiol.* 89 383–389. 10.1016/j.pneurobio.2009.10.002 19819292

[B126] Taniguchi N. Miyata M. Yachiku S. Kaneko S. Yamaguchi S. Numata A. (2002). A study of micturition inducing sites in the periaqueductal gray of the mesencephalon. *J. Urol.* 168 1626–1631. 10.1016/S0022-5347(05)64532-6 12352469

[B127] Taylor N. E. Pei J. Zhang J. Vlasov K. Y. Davis T. Taylor E. (2019). The role of glutamatergic and dopaminergic neurons in the periaqueductal gray/dorsal raphe: Separating analgesia and anxiety. *Eneuro* 6:ENEURO.0018-18.2019. 10.1523/ENEURO.0018-18.2019 31058210 PMC6498422

[B128] Tovote P. Esposito M. S. Botta P. Chaudun F. Fadok J. P. Markovic M. (2016). Midbrain circuits for defensive behaviour. *Nature* 534 206–212. 27279213 10.1038/nature17996

[B129] Trevizan-Baú P. Dhingra R. R. Furuya W. I. Stanić D. Mazzone S. B. Dutschmann M. (2021a). Forebrain projection neurons target functionally diverse respiratory control areas in the midbrain, pons, and medulla oblongata. *J. Comp. Neurol.* 529 2243–2264. 10.1002/cne.25091 33340092

[B130] Trevizan-Baú P. Furuya W. I. Mazzone S. B. Stanić D. Dhingra R. R. Dutschmann M. (2021b). Reciprocal connectivity of the periaqueductal gray with the ponto-medullary respiratory network in rat. *Brain Res.* 1757:147255. 10.1016/j.brainres.2020.147255 33515533

[B131] Vaaga C. E. Brown S. T. Raman I. M. (2020). Cerebellar modulation of synaptic input to freezing-related neurons in the periaqueductal gray. *Elife* 9:e54302 10.7554/eLife.54302 32207681 PMC7124251

[B132] Vander Weele C. M. Siciliano C. A. Matthews G. A. Namburi P. Izadmehr E. M. Espinel I. C. (2018). Dopamine enhances signal-to-noise ratio in cortical-brainstem encoding of aversive stimuli. *Nature* 563 397–401. 10.1038/s41586-018-0682-1 30405240 PMC6645392

[B133] Vaughn E. Eichhorn S. Jung W. Zhuang X. Dulac C. (2022). Three-dimensional interrogation of cell types and instinctive behavior in the periaqueductal gray. *bioRxiv [Preprint].* 10.1101/2022.06.27.497769.

[B134] Vázquez-León P. Miranda-Páez A. Valencia-Flores K. Sánchez-Castillo H. (2023). Defensive and emotional behavior modulation by serotonin in the periaqueductal gray. *Cell Mol. Neurobiol.* 43 1453–1468. 35902460 10.1007/s10571-022-01262-z PMC11412428

[B135] Vieira-Rasteli E. B. de Paula B. B. de Paiva Y. B. Coimbra N. C. Leite-Panissi C. R. A. (2018). Restricted lesions of the ventrolateral or dorsal columns of the periaqueductal gray promotes distinct effects on tonic immobility and defensive analgesia in guinea pigs. *Physiol. Behav.* 194 538–544. 30003893 10.1016/j.physbeh.2018.07.003

[B136] Villela D. C. da Silva L. G. Jr. Fontes M. A. (2009). Activation of 5-HT receptors in the periaqueductal gray attenuates the tachycardia evoked from dorsomedial hypothalamus. *Auton. Neurosci.* 148 36–43. 10.1016/j.autneu.2009.02.004 19303372

[B137] Wang L. Chen I. Z. Lin D. (2015). Collateral pathways from the ventromedial hypothalamus mediate defensive behaviors. *Neuron* 85 1344–1358. 10.1016/j.neuron.2014.12.025 25754823 PMC4368499

[B138] Wang W. Schuette P. J. La-Vu M. Q. Torossian A. Tobias B. C. Ceko M. (2021a). Dorsal premammillary projection to periaqueductal gray controls escape vigor from innate and conditioned threats. *eLife* 10:e69178. 10.7554/eLife.69178 34468312 PMC8457830

[B139] Wang W. Schuette P. J. Nagai J. Tobias B. C. Cuccovia V. Reis F. M. (2021b). Coordination of escape and spatial navigation circuits orchestrates versatile flight from threats. *Neuron* 109 1848–1860.e8 10.1016/j.neuron.2021.03.033 33861942 PMC8178241

[B140] Wang Y. Q. Liu W. Y. Li L. Qu W. M. Huang Z. L. (2021c). Neural circuitry underlying REM sleep: A review of the literature and current concepts. *Prog. Neurobiol.* 204:102106. 10.1016/j.pneurobio.2021.102106 34144122

[B141] Webb E. K. Huggins A. A. Belleau E. L. Taubitz L. E. Hanson J. L. deRoon-Cassini T. A. (2020). Acute posttrauma resting-state functional connectivity of periaqueductal gray prospectively predicts posttraumatic stress disorder symptoms. *Biol. Psychiatry Cogn. Neurosci. Neuroimaging* 5 891–900. 10.1016/j.bpsc.2020.03.004 32389746 PMC7483700

[B142] Weber F. Chung S. Beier K. T. Xu M. Luo L. Dan Y. (2015). Control of REM sleep by ventral medulla GABAergic neurons. *Nature* 526 435–438. 26444238 10.1038/nature14979 PMC4852286

[B143] Weber F. Hoang Do J. P. Chung S. Beier K. T. Bikov M. Saffari Doost M. (2018). Regulation of REM and non-REM sleep by periaqueductal GABAergic Neurons. *Nat. Commun.* 9:354. 10.1038/s41467-017-02765-w 29367602 PMC5783937

[B144] Westermann B. Lotze M. Varra L. Versteeg N. Domin M. Nicolet L. (2022). When laughter arrests speech: fMRI-based evidence. *Philos. Trans. R Soc. Lond. B Biol. Sci.* 377:20210182. 10.1098/rstb.2021.0182 36126674 PMC9489293

[B145] Wilson-Poe A. R. Wiese B. Kibaly C. Lueptow L. Garcia J. Anand P. (2021). Effects of inflammatory pain on CB1 receptor in the midbrain periaqueductal gray. *Pain Rep.* 6:e897. 10.1097/PR9.0000000000000897 PMC7939232 33693301

[B146] Xie L. Wu H. Chen Q. Xu F. Li H. Xu Q. (2023). Divergent modulation of pain and anxiety by GABAergic neurons in the ventrolateral periaqueductal gray and dorsal raphe. *Neuropsychopharmacology* 48 1509–1519. 36526697 10.1038/s41386-022-01520-0 PMC10425368

[B147] Xie Z. Gu H. Huang M. Cheng X. Shang C. Tao T. (2022). Mechanically evoked defensive attack is controlled by GABAergic neurons in the anterior hypothalamic nucleus. *Nat. Neurosci.* 25 72–85. 10.1038/s41593-021-00985-4 34980925

[B148] Yin J. B. Liang S. H. Li F. Zhao W. J. Bai Y. Sun Y. (2020). dmPFC-vlPAG projection neurons contribute to pain threshold maintenance and antianxiety behaviors. *J. Clin. Invest.* 130 6555–6570. 10.1172/JCI127607 32841213 PMC7685740

[B149] Yu H. Xiang X. Chen Z. Wang X. Dai J. Wang X. (2021). Periaqueductal gray neurons encode the sequential motor program in hunting behavior of mice. *Nat. Commun.* 12:6523. 10.1038/s41467-021-26852-1 34764279 PMC8586038

[B150] Yu W. Pati D. Pina M. M. Schmidt K. T. Boyt K. M. Hunker A. C. (2021). Periaqueductal gray/dorsal raphe dopamine neurons contribute to sex differences in pain-related behaviors. *Neuron* 109 1365–1380.e5. 10.1016/j.neuron.2021.03.001 33740416 PMC9990825

[B151] Zangrossi H. Jr. Graeff F. G. (2014). Serotonin in anxiety and panic: Contributions of the elevated T-maze. *Neurosci. Biobehav. Rev.* 46(Pt 3), 397–406. 10.1016/j.neubiorev.2014.03.007 24657635

[B152] Zare A. Jahanshahi A. Meriaux C. Steinbusch H. W. van Koeveringe G. A. (2018). Glutamatergic cells in the periaqueductal gray matter mediate sensory inputs after bladder stimulation in freely moving rats. *Int. J. Urol.* 25 621–626. 10.1111/iju.13562 29577439

[B153] Zare A. Jahanshahi A. Rahnama’i M. S. Schipper S. van Koeveringe G. A. (2019). The role of the periaqueductal gray matter in lower urinary tract function. *Mol. Neurobiol.* 56 920–934. 29804231 10.1007/s12035-018-1131-8 PMC6400878

[B154] Zhang G. W. Shen L. Tao C. Jung A. H. Peng B. Li Z. (2021). Medial preoptic area antagonistically mediates stress-induced anxiety and parental behavior. *Nat. Neurosci.* 24 516–528. 10.1038/s41593-020-00784-3 33526942 PMC8328037

[B155] Zhang Y. Huang X. Xin W. J. He S. Deng J. Ruan X. (2023). Somatostatin neurons from periaqueductal gray to medulla facilitate neuropathic pain in male mice. *J. Pain* 24 1020–1029. 10.1016/j.jpain.2023.01.002 36641028

[B156] Zhao Z. D. Chen Z. Xiang X. Hu M. Xie H. Jia X. (2019). Zona incerta GABAergic neurons integrate prey-related sensory signals and induce an appetitive drive to promote hunting. *Nat. Neurosci.* 22 921–932. 10.1038/s41593-019-0404-5 31127258

[B157] Zhao Z. D. Zhang L. Xiang X. Kim D. Li H. Cao P. (2023). Neurocircuitry of predatory hunting. *Neurosci. Bull.* 39 817–831. 10.1007/s12264-022-01018-1 36705845 PMC10170020

[B158] Zhong P. Zhang Z. Barger Z. Ma C. Liu D. Ding X. (2019). Control of non-REM sleep by midbrain neurotensinergic neurons. *Neuron* 104 795–809.e6. 10.1016/j.neuron.2019.08.026 31582313

